# Neurotropic Astroviruses in Animals

**DOI:** 10.3390/v13071201

**Published:** 2021-06-23

**Authors:** Nicole Wildi, Torsten Seuberlich

**Affiliations:** Division of Neurological Sciences, Vetsuisse Faculty, University of Bern, 3012 Bern, Switzerland; nicole.wildi@vetsuisse.unibe.ch

**Keywords:** astrovirus, non-suppurative encephalitis, neurological disease

## Abstract

Astrovirus infections are among the main causes of diarrhea in children, but their significance for animal health has remained underestimated and largely unknown. This is changing due to the increasing amount of newly identified neurotropic astroviruses in cases of nonsuppurative encephalitis and neurological disease in humans, pigs, ruminant species and minks. Neurological cases in ruminants and humans usually occur sporadically and as isolated cases. This contrasts with the situation in pigs and minks, in which diseases associated with neurotropic astroviruses are endemic and occur on the herd level. Affected animals show neurological signs such as mild ataxia to tetraplegia, loss of orientation or trembling, and the outcome is often fatal. Non-suppurative inflammation with perivascular cuffing, gliosis and neuronal necrosis are typical histological lesions of astrovirus encephalitis. Since astroviruses primarily target the gastrointestinal tract, it is assumed that they infect the brain through the circulatory system or retrograde following the nerves. The phylogenetic analysis of neurotropic astroviruses has revealed that they are genetically closely related, suggesting the presence of viral determinants for tissue tropism and neuroinvasion. In this review, we summarize the current knowledge on neurotropic astrovirus infections in animals and propose future research activities.

## 1. Introduction

Virus-induced encephalitis is a major health problem worldwide in animals and humans. Some of the associated viruses, such as rabies virus, Japanese encephalitis virus, Aujeszky’s disease virus, Borna virus and West-Nile virus, are transmitted between animals and humans, posing a significant threat to public health. In humans and animals, there are still large numbers of viral encephalitis cases with an unknown etiology [1,2,3,4]. Since high-throughput sequencing (HTS) techniques and the bioinformatics analysis of HTS data for virus identification and discovery have become widely available, previously unresolved encephalitis cases could be attributed to brain infections with known as well as novel viruses [5,6,7]. Among these discoveries were neurotropic astroviruses (NT-AstV) in humans and a series of animal species [8,9,10,11].

Astroviruses were first described in 1975 in human feces [12]. They are small (~35 nm), non-enveloped, positive-sense, single-stranded RNA viruses. The genetic diversity of astroviruses is huge, and many animal species have been identified as hosts. Taxonomically, the family *Astroviridae* is currently divided into two genera, *Mamastrovirus* and *Avastrovirus*, with mammals and birds as natural hosts, respectively. Within the genus *Mamastrovirus,* 19 genotype species (*Mamastrovirus [MAstV] 1-19*) are recognized by the International Committee on Taxonomy of Viruses [13], and an additional 14 genotype species (*MAstV 20-33*) have been proposed. However, the spectrum of astrovirus strains discovered in mammalian species has massively expanded over the past years. Most of these new strains currently remain unclassified, and many of them will likely need to be assigned to new genotype species. Thus, the current taxonomy is outdated and urgently needs revision. Moreover, astroviruses beyond the defined genera were discovered in fish, amphibia and reptiles [14]. Genetically, these viruses cluster apart from members of the genera *Mamastro- and Avastroviruses,* indicating that astroviruses have adapted to different types of vertebrate animals, leading to the hypothesis that they infect invertebrate animals as well. While most members of the genus *Avastrovirus* are associated with disease and often lead to systemic infections [15,16], astrovirus infections in mammalians are mostly restricted to the gastrointestinal tract (GIT) and not associated with severe disease. A prominent exception are infections with classical human astroviruses (*MAstV 1*), which are a common cause of acute diarrhea in infants [17]. The recent discovery of astroviruses in the brains of animals—i.e., minks, cattle, sheep, pigs, a muskox and an alpaca—as well as in humans with neurological disease and encephalitis has changed our view on the involvement of these viruses in non-enteric diseases. Here, we summarize the current knowledge on NT-AstV in animals, point to gaps in our understanding of disease mechanisms and indicate future directions of research.

## 2. Molecular Biology of Astroviruses

**Figure 1:**
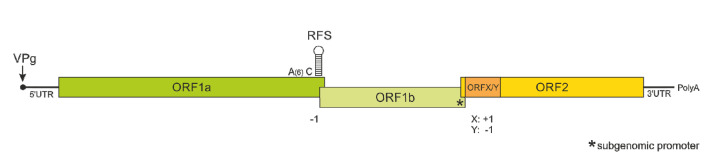
Schematic representation of the astrovirus genome. The ribosomal frameshift signal between ORF 1a and ORF 1b (RFS) is located downstream of a slippery sequence A(6)C, resulting in an incomplete −1 frameshift and the translation of the nonstructural proteins nsp1a and nsp1ab, respectively. A conserved subgenomic promoter sequence (*) leads to the translation of the ORF2 during viral replication from a subgenomic RNA. Additionally, the translation of ORFX encoded in the +1 frame or of ORF Y encoded in the −1 frame with reference to the ORF2 is proposed being translated by a ribosomal leaky scanning mechanism.

The genome of astroviruses is between ~6100 and ~7700 nucleotides (nt) in size and contains three open reading frames (ORFs). ORF1a encodes for non-structural proteins, ORF1b for the RNA-dependent RNA polymerase and ORF2 for capsid proteins [17]. In addition, members of some astrovirus genotype species show a fourth ORF (ORFX or ORFY), which overlaps with ORF2 and encodes a protein with a viroporin-like activity [18]. The presence or absence of these additional ORFs is apparently not associated with the capacity of the virus to invade the nervous system, because some NT-AstV contain ORFX (bovine astrovirus (BoAstV) CH13/NeuroS1) or ORFY (mink astrovirus (MiAstV) SMS) and some do not (porcine astrovirus (PoAstV-3)). Two untranslated regions (UTRs) at the 5′ and 3′ end flank the genome. The 5′ UTR is linked to a viral genome-linked protein (VPg) and it is reported to range from 14 nt to 51 nt [19]. Generally, all reported NT-AstV have a CCAAA pentamer at the 5′ terminus. In some reported genome sequences, this is missing, probably due to incomplete sequencing. The 3’ UTR is followed by a poly-A-tail. Common characteristics of all members of the *Astroviridae* are the presence of a ribosomal frameshift signal (RFS) between the ORF1a and ORF1b, resulting in the translation of the non-structural polyprotein nsp1ab, and a subgenomic promoter region upstream of the start codon of the ORF2. This subgenomic (sg) promoter region is highly conserved in NT-AstV [19] (Figure 1).

## 3. Neuropathology of Astrovirus-Associated Encephalitis

**Figure 2:**
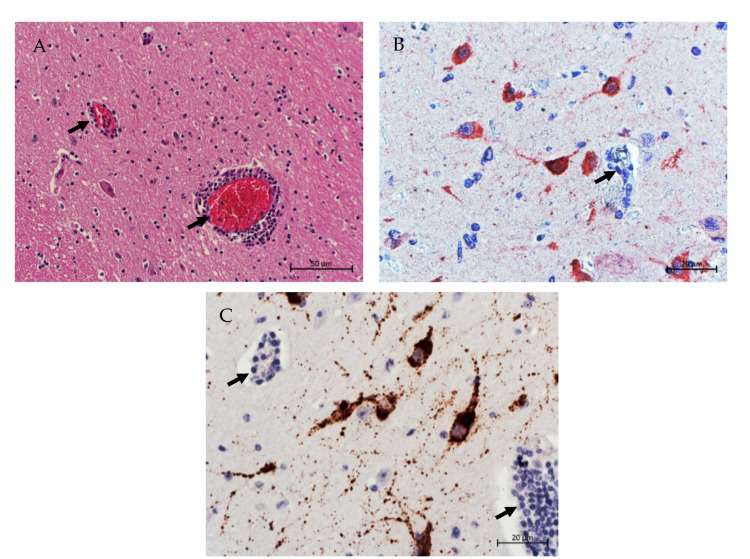
Neuropathology of astrovirus-associated encephalitis in the mesencephalon of a cattle (female, 1 ¼ years, strain bovine astrovirus CH13/NeuroS1 (BoAstV CH13/NeuroS1)). (**A**) HE-staining, showing mild hypercellularity (gliosis) and perivascular cuffs involving mononuclear leukocytes. (**B**) Demonstration of viral antigen in neurons by immunohistochemistry (IHC) with a polyclonal antibody against the capsid protein of BoAstV-CH13/NeuoS1 [32] (red labeling). (**C**) Demonstration of viral RNA in neurons by in situ hybridization (ISH) with a probe targeting the ORF2 of BoAstV-CH13/NeuroS1 (brown labeling). The arrows point to perivascular cuffs, and scale bars are presented at the bottom right.

The main type of histologic lesion associated with astrovirus infection of the central nervous system (CNS) is non-suppurative encephalitis/encephalomyelitis, which includes the perivascular and neuroparenchymal infiltration of inflammatory cells, predominantly lymphocytes, histiocytes and fewer plasma cells and gliosis (Figure 2A). These lesions are more often seen in the gray rather than the white matter, and there is frequent scattered neuronal necrosis. Occasionally, the inflammation also involves the meninges (meningitis). This pattern of lesions was observed in all affected species [10,11,20,21,22,23,24] and is typical for virus-induced encephalitis [25]. Inclusion bodies have not been reported. In ruminants, pigs and minks, the lesions were often most severe in the brainstem [8,9,26,27,28]. If the spinal cord was available, which was the case especially for pigs, minks and cattle, it was generally strongly affected as well [8,9,26,29]. In the spinal cord, the ventral horns often showed typical inflammatory lesions, and in severe cases, the inflammation progressed into the white matter and leptomeninges [26]. Cell tropism appears to be variable between different NT-AstV. In human cases, viral protein and viral RNA were detected in astrocytes [11] and neurons [30], while in ruminants and pigs, mainly neurons and only occasionally glial cells were affected (Figure 2B,C) [21,24,26,31].

## 4. Neurotropic Astroviruses in Different Animal Species

### 4.1. Neurotropic Astrovirus in Minks

**Figure 3:**
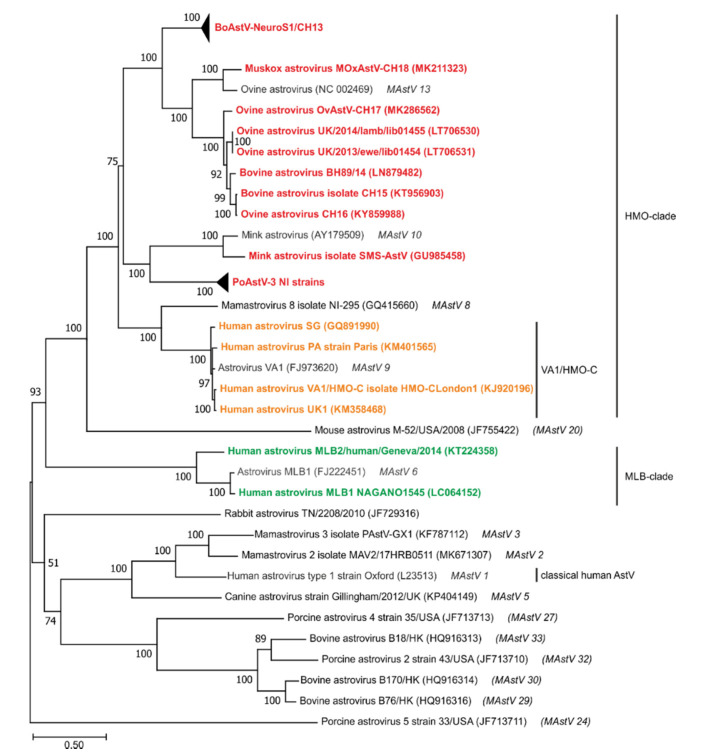
Phylogenetic analysis of available full-length genomes of neurotropic astroviruses. Officially recognized Mamastrovirus (MAstV) genotype species are indicated in italics, whereas the provisional MAstV genotype species are in parentheses. Strains detected in brain tissues in association with encephalitis are highlighted for humans (orange and green) and animals (red). Strains included in the analysis were selected based on the former classification with the available full-length reference genomes [61] and the available full-length NT-AstV strains. Sequences were aligned with MUSCLE [62], and the tree was constructed with MEGA X [63] using the Maximum Likelihood method and General Time Reversible model [64] with 1000 bootstrap replications. The bar indicates nucleotide sequence p-distances. The BoAstV CH13/NeuroS1 clade includes strains with GenBank accession numbers KF233994, KM03579, KX266901–KX266908, KT956903, LC341267 and LN879482. The PoAstV-3 clade includes strains with GenBank accession numbers KY940545, KY073229, KY073231, KY073232 and JX556691.

Between 2000 and 2012, a previously unknown disease emerged in farmed mink kits in Denmark, Sweden and Finland. Typical neurological signs included seizures, ataxia, salivation and shaking, and the condition was designated “shaking mink syndrome” (SMS). Affected animals were not restricted to specific litters but occurred randomly in different parts of the farms. According to the farmers’ observations, the morbidity was 0.2% to 0.8% and the case fatality rate was 27% to 28%. To investigate an infectious origin, three mink kits were infected experimentally with the brain homogenate of diseased animals. All three experimentally infected mink kits developed neurological signs, and the disease was shown to be transmissible. Additionally, clinical signs and histological lesions—i.e., non-suppurative encephalitis—were similar in naturally and experimentally infected animals. At that point in time, the etiology of this disease remained unresolved [23]. Later, brain tissue samples were subjected to HTS, and a new astrovirus was identified and designated as SMS-AstV (GenBank accession GU985458). SMS-AstV is phylogenetically closely related to an already known enteric mink astrovirus (MiAstV, *MAstV 10*) (Figure 3), which is associated with pre-weaning diarrhea in mink kits [8,33]. These results showed for the first time that astroviruses could invade the CNS in animals, causing encephalitis and fatal neurological diseases with significant losses on the farm level.

A very recent study from Poland reported a similar disease on two farms, on which mink kits suffered from neurological signs as mentioned above. Brain and intestinal tissues of six animals were examined by RT-PCR for MiAstV. All brain and intestinal samples were positive. The obtained amplicon sequences were 100% identical to each other and >95% identical to MiAstV (*MAstV 10*) and SMS-AstV [34], suggesting that in the case of SMS, the virus is not exclusively targeting the nervous tissue but also the GIT. Unfortunately, a pathological examination of these kits was not reported, so whether the neurological signs correlated with encephalitis or lesions in other organs remained undetermined.

### 4.2. Neurotropic Astroviruses in Pigs

The first report of astroviruses associated with neurological disease in pigs was in 2014 [35]. Blomström et al. investigated piglets that showed congenital tremor (subtype AII), which can cause important losses in intense pig production [35]. In diseased piglets, porcine astroviruses and porcine circovirus-2 were detected in the CNS by RT-PCR. The amplicons of the astrovirus-positive samples were sequenced, and two different strains of porcine astroviruses were identified: *MAstV 31* (PoAstV-2) and *MAstV 24* (PoAstV-5), respectively. However, similar sequences were also found in brain tissues of healthy piglets, questioning the causal relationship between astrovirus infection and congenital tremor.

In 2017, two studies simultaneously reported for the first time a clear association of astrovirus infection of the CNS, encephalitis and neurological disease in pigs from three farms in Hungary and a multi-site farm in the USA [20,36]. The clinical signs ranged from mild posterior paraparesis to severe tetraplegia, and occasionally the animals showed seizures [36]. In Hungary, the disease mostly occurred in approximately one-month-old weaned pigs, whereas in the USA, mainly sows and older piglets were affected. The case-fatality rate in the USA was 75–100%. In both studies, viral sequences were generated from brain tissue (Table 1). These sequences clustered to the already known PoAstV-3 strain US-MO123 (*MAstV 22*), which was obtained from the feces of a diarrheic pig [37]. Both studies confirmed the presence of PoAstV-3 RNA by RT-qPCR and/or ISH in different CNS tissues. Additional cases were identified pro- and retro-spectively in two follow-up studies in the USA [26,38]. In the cases reported from Hungary, the viral RNA was constantly detected in different extraneuronal tissues including the GIT of affected animals by RT-PCR (Table 1), suggesting that PoAstV-3 can cause systemic infections. This was, however, not the case for the animals reported from the USA. This divergence may be related to the diagnostic sensitivity of the RT-PCR protocol applied, to different stages of disease at the time of sampling or possibly to age-related differences in disease pathogenesis.

The fecal shedding of PoAstV-3 varies greatly among different farms. Some authors mention very high proportions [39,40] and others very low proportions [41,42] of virus-positive animals. On farms with known PoAstV-3 CNS infections, the detection of viral RNA in GIT samples of diseased animals was rare [20,26,36,38]. On the other hand, PoAstV-3 RNA was also detected in nervous tissues (myenteric plexus neurons, by ISH), as well in enterocytes, lamina propria, Peyer’s patches and mesenteric lymph nodes in healthy animals from farms without a history of PoAstV-3 associated encephalitis [40]. Taken together, with the limited data available, there is no clear association between the presence of PoAstV-3 in the GIT and in the nervous tissue, and the pathogenesis of disease—in particular, the route of neuroinvasion—remains unknown.

### 4.3. Neurotropic Astroviruses in Cattle

A novel astrovirus (BoAstV NeuroS1) was discovered in a crossbreed steer showing neurologic signs and non-suppurative encephalitis in the USA in 2013 by HTS-based metagenomics. The same astrovirus strain was then detected retrospectively in three other cattle by RT-PCR [9]. A few months later, in an independent virus discovery study, a very similar astrovirus strain, BoAstV CH13, with >95% sequence similarity of the viral genome to BoAstV NeuroS1, was identified in five cows with non-suppurative encephalitis in Switzerland [10]. These findings triggered a series of pro- and retro-spective investigations into the etiology of unresolved cases of non-suppurative encephalitis in cattle. To date, there are >50 confirmed cases of brain infection in cattle with BoAstV NeuroS1/CH13 or very similar strains reported in the literature from Europe, the Americas and Asia, and early positive cases date back to the 1960s (Table 2) [9,27,28,29,31,43,44,45,46,47].

A second NT-AstV, distinct from BoAstV CH13/NeuroS1, was detected in two cows: one in Germany (BoAstV BH89/14) [48] and one in Switzerland [49]. Although genetically almost identical (Figure 2), the strains were named differently by the authors. All four NT-AstV strains in cattle—BoAstV NeuroS1, BoAstV-CH13, BoAstV BH89/14 and BoAstV CH15—are most similar to Ovine Astrovirus 1 (OvAstV-1, *MAstV 13*), originally detected in lambs with gastrointestinal signs [50], with the BoAstV-BH89/14 and BoAstV CH15 being more closely related to OvAstV-1 than BoAstV NeuroS1/CH13.

For bovine animals, anamnestic information is often sparse, and the knowledge on specific neurological clinical signs is often very limited. However, in 17 cases, a more detailed description of the neurologic signs is available. One study compared the clinical signs of nine different cases in Switzerland [51]. The signs ranged from solely aggressive behavior to lateral recumbency with severe cranial nerve deficits (e.g., reduced menace response, reduced sensitivity). Moving disorders were reported in each case, except for one. These findings are consistent with the signs reported from the cases in the USA [9], Italy [45], Japan [29], Uruguay [43] and Germany [48]. Fever was present in three cases, [29,51], and one animal exhibited coughing [51].

All the viral sequences were obtained from CNS samples, and in most cases, an IHC and/or ISH was performed to confirm the presence of NT-AstV in the brain tissue (Table 2). Besides the detection of NT-AstV in CNS samples, one cow showed a dorsal root ganglioneuritis [9], and another one an inflammation of the trigeminal ganglion [48]. Viral RNA of strain BoAstV BH89/14 was further detected in the spleen, liver and the pancreatic tissue by RT-qPCR and ISH, but not in the heart, muscles and the lungs. The kidney tissue was negative by RT-qPCR but stained positive in ISH [48]. Screening of lung, liver, spleen, kidney, mesenteric lymph node and GIT tissues of the case in Japan by RT-qPCR remained negative as well.

**Table 1:** Reports on porcine astrovirus (PoAstV) 3 associated encephalitis cases in pigs and virus detection in non-neuronal tissues of affected animals. Only animals with histopathological lesions of non-suppurative encephalitis and PoAstV-3 detection by RT-PCR in the brain were included.

Report Country/Reference	No. of Cases	Respiratory Tract ^1^	Lymphatic Tissue	Serum	Liver	GIT ^2^	Heart
Hungary [20]	8	4/4	3/4	2/2	2/2	2/4	2/2
USA [36]	4	0/4	nd	0/4	0/4	0/4	nd
USA [26]	22	0/19	1/12	nd	0/18	2/15	0/16
USA [38]	3	0/2	0/2	nd	0/2	0/2	0/2

Numbers are given as animals that tested positive/total tested animals; ^1^: including nasal swabs; ^2^: including fecal samples. Abbreviations: GIT; gastrointestinal tract; nd; not done.

**Table 2:** Reports on astroviruses associated with encephalitis in ruminant species.

Host	Report Country [References]	No. of Cases	Histopathology	ISH/IHC	Strain	GenBank Accession Number ^1^
Cattle	USA [9]	4 ^1^	+	+	BoAstV NeuroS1	KF233994
Cattle	Switzerland [10,28,31,46]	47 ^1,2^	+	+	BoAstV CH13	KM03579/KX266901 to KX266908
Cattle	Switzerland [49]	2 ^2^	+	+	BoAstV CH15	KT956903
Cattle	Uruguay [43]	1	+	+	BoAstV-Neuro-Uy	MK386569
Cattle	Canada [27,47]	6 ^1^	+	+	BoAstV NeuroS1/CH13	KY614055/KY614056
Cattle	Italy [45]	1	+	+	BoAstV PE3373/2019/Italy	MN46146
Cattle	Japan [29]	1	+	nd	KagoshimaSR28-462Brain	LC341267
Cattle	Germany [48]	1	+	+	BoAstV BH89/14	LN879482
Sheep	Switzerland [22,52]	2	+	+	OvAstV CH16 OvAstV CH17	KY859988 MK286562
Sheep	United Kingdom [53]	2	+	+	OvAstV UK/2013/ewe/lib01454 OvAstV UK/2014/lamb/lib01455	LT706531 LT706530
Muskox	Switzerland [21]	1	+	+	MOxAstV CH18	MK211323
Alpaca	Switzerland [24]	1	+	+	BoAstV-CH13/NeuroS1	-

^1^: Not all samples were sequenced; ^2^: in one brain sample sequences of BoAstV-CH13 and BoAstV-CH15 strain were detected. Abbreviations: +: positive, nd: not done; (S): Sanger sequencing; ISH: in situ hybridization; IHC: immunohistochemistry.

### 4.4. Neurotropic Astroviruses in Sheep

In total, four astrovirus-associated encephalitis cases have been discovered in sheep to date: two in Switzerland [22,52] and two in North Wales [53]. The affected sheep in North Wales exhibited trembling and moving disorders [53]. In Switzerland, one ewe showed ataxia and fever, and a foreign body was stuck in the esophagus [22]. In all cases, the RNA was extracted from brain tissue and sequenced by HTS [22,53] or Sanger sequencing [52]. The screening of other organs by RT-qPCR in affected sheep showed some positive results in GIT samples and trigeminal ganglion, but not in the lymph node or spleen [53]. Besides the CNS lesions, a ganglionitis was present in the British cases [53]. Laboratory diagnostic procedures and the number of cases are summarized in Table 2.

The obtained sequences from the CNS clustered very closely to the earlier described BoAstV CH15 and BoAstV BH 89/14 strains (Figure 3). Indeed, in Switzerland, the newly identified strains (OvAstV CH16 and OvAstV CH17) were confirmed in the CNS by an IHC protocol using polyclonal antisera generated against the capsid protein of BoAstV-CH15 [22,52].

### 4.5. Neurotropic Astrovirus in a Muskox 

Boujon et al. [21] discovered a novel astrovirus, named muskox astrovirus CH18 (MOxAstV CH18), in archived formalin-fixed paraffin-embedded brain tissues of a muskox (*Ovibos moschatus*) diagnosed with severe non-suppurative encephalitis in 1982. The six-year-old muskox was from the Bern Animal Park, Switzerland and showed paraparesis firstly in the hind limbs. The neurological status rapidly decreased until the animal could not walk due to tetraplegia, and finally, it was euthanized. In 2018, the screenings of fecal samples from muskoxen of the same animal park by RT-qPCR for MOxAstV CH18 RNA were all negative.

The presence of the virus in brain tissue was confirmed by two different IHC methods: RT-qPCR and HTS. Again, the two IHC protocols applied were originally developed for the detection of BoAstV-CH13/NeuroS1 and BoAstV-CH15, respectively, but revealed a clear labeling of viral proteins in neurons due to the cross-reactivity of the polyclonal antisera with MOxAstV-CH18 antigens. The phylogenetic analysis on the full-length genome showed the closest relationship to OvAstV 1 [54,55] (Figure 3). Whether this animal had been in contact with other ruminant species, as a potential source of infection, could not be evaluated due to the elapsed time.

### 4.6. Neurotropic Astrovirus in an Alpaca

Very recently (2020), the first reported case of astrovirus-associated encephalitis in an alpaca was reported [24]. The alpaca was eight years old and presented to the Clinic for Ruminants at the University of Bern because of a decreased general condition, anorexia, reduced gastrointestinal peristalsis and neurological signs (e.g., ataxia, intermittent tremor and absence of menace response in the left eye). Therapeutic interventions were unsuccessful, and two days later, the animal was euthanized and submitted for pathologic examination. The histopathological examination of the CNS revealed non-suppurative polioencephalomyelitis and prompted additional testing. IHC, ISH and RT-qPCR designed for BoAstV CH13/NeuroS1 were positive on CNS tissue including the spinal cord. Additionally, IHC of the kidney, heart, liver and uterus was performed and scored negative. CNS samples were sequenced by HTS, and all results confirmed that the alpaca was infected with the BoAstV-NeuroS1/CH13 strain. According to the livestock owner, this animal had not been in close contact with other domestic animals. The other three alpacas from the same farm did not show neurological signs. Fecal and nasal swabs and blood samples were negative by RT-qPCR three months after the diseased alpaca had died [24].

## 5. Phylogeny

Phylogenetically, NT-AstV identified in animals all belong to the so-called HMO-AstV clade (human–mink–ovine). In humans astroviruses of the MLB-AstV clade (MLB: Melbourne), Human Astrovirus 1 (HuAstV-1, *MAstV 1*) [56] and Human Astrovirus 4 (HuAstV-4, *MAstV 1*) [57] were also found to have an association with encephalitis. The HMO clade originally contained newly discovered astrovirus strains from humans (HuAstV VA1) [58] and mink feces samples (MiAstV, *MAstV 10*), which showed genetic similarity to OvAstV 1 (*MAstV 13*). Consequently, this clade was termed HMO.

Taxonomically, NT-AstV in pigs clearly belong to PoAstV-3 (proposed genotype species *MAstV 22*) and the NT-AstV SMS in minks is a member of *MAstV 10.* Ruminant NT-AstV strains show the greatest similarity to OvAstV-1, and several authors have proposed that they should be classified as *MAstV 13* [29,43,59].

It is striking that astroviruses detected in brain tissues in animals with encephalitis (ruminants, minks and pigs) and humans are genetically closely related, while most strains detected in the GIT of these species are much more diverse [60]. It is tempting to speculate that these viruses share common mechanisms of host-invasion with a particular tropism for the nervous system. If there are such pathogen-encoded determinants, this still needs to be resolved. Furthermore, the close genetic (and antigenetic) association of these strains raises concerns about the potential interspecies transmission of these virus strains. This seems to be the case in ruminants (Figure 3) but would be of major concern in case of zoonotic transmission. However, epidemiological evidence for such scenarios is not yet available.

## 6. Prophylaxis and Therapeutics

To date, vaccination is not available for astroviruses. As a possible approach to reduce the amount of viral load and affected animals, the cleaning and disinfection of pens has resulted in improvements in pig herds, but no clearing of the virus [38]. However, in PoAstV-3 affected farms in Hungary, it was possible to reduce the numbers of diseased pigs from 30–40 to 1–2 cases per month [20] by these means. It is known that astroviruses are quite stable in the environment and are not very sensitive to many disinfectants [65,66,67]. Further studies on the efficacy of practicable disinfection methods are needed.

As therapeutic options, besides symptomatic and support therapy, no effective antiviral treatment against NT-AstV infection is available. It is not known how efficient broad-spectrum antiviral drugs are, since there are no in vitro or in vivo models for most of the NT-AstV strains. Some in vitro studies showed a decreased replication activity of the virus when broad spectrum antiviral and/or immunomodulatory drugs were used on astrovirus-infected cell cultures [68,69,70,71,72,73,74]. One study showed that nitazoxanide reduces clinical signs of diarrhea and the amount of turkey astrovirus Strain 2 (*Avastrovirus*) RNA in the feces of experimentally infected turkeys. However, nitazoxanide was administered four days prior to infection [73]. In fact, it is questionable how realistic antiviral therapies would be for livestock, where food safety regulations need to be considered.

## 7. Pathogenesis

To date, there is still a lack of knowledge regarding the pathogenesis and the development of neurological symptoms due to an astrovirus infection. The NT-AstV strains clustering in the HMO-clade show similar histopathological lesions and clinical symptoms. In humans, the neuroinvasion of astroviruses is often linked to an immunocompromised status, which leads to a fatal outcome of NT-AstV infection [11,30,75,76]. In animals, it is difficult to define immune statuses under field conditions in detail. It is known that the environment and animal density on farms can be stressors that lead to an immunocompromised status. Probably, genetic determinants in the viral and/or host genome, together with an underlying disease, co-infection or unrecognized stressor, are responsible for the viral invasion of the brain. This view is supported by the finding that astrovirus-associated encephalitis was found to be endemic in mink and pig farms where animals are kept under intense production conditions. On the other hand, astrovirus-associated encephalitis of ruminants seems to occur sporadically and as isolated cases, with no obvious stressor related to the production system.

There is evidence for NT-AstV being transmitted fecal–orally, as is known from enteric astroviruses. NT-AstV of bovines, pigs, ovines and minks were detected in intestinal/fecal samples of diarrhetic or healthy animals [34,37,40,77]. Possibly, NT-AstV invade the CNS from the GIT retrograde along the peripheral nerves or the circulatory system, since there are cases in which NT-AstV infection induced dorsal root ganglionitis [9,48,53] or could be detected by ISH in the myenteric plexus and mesenteric lymph nodes [40]. On a different plane, the detection of PoAstV-3 in nasal swabs of pigs [20] and of HuAstV VA1 in nasopharyngeal specimen of a febrile child, suffering from a respiratory disease [78] indicates a second possible site of virus entry. Whether virus transmission and the invasion of the host involves other organ systems, such as the reproductive tract, and causes a systemic infection and/or viremia remains to be investigated.

## 8. Conclusions

Although HTS technologies have allowed some non-suppurative encephalitis cases to be attributed to infection with NT-AstV in recent years, little is known about the pathogenesis and epidemiology of the disease induced by these viruses. HTS is highly useful to detect and identify NT-AstV in brain tissue without any prior knowledge of their genome or protein structure and is increasingly applied in routine diagnostics. Certainly, the application of HTS (or astrovirus specific diagnostic techniques) in unclear disease situations with indications of viral infections will lead to a better understanding of the role of astroviruses in healthy and diseased humans and animals.

Nevertheless, the challenge in clinical NT-AstV research is not only the diagnostic capacity but rather the availability of appropriate samples. Obtaining brain tissue from affected individuals is often difficult, as this requires biopsy or post-mortem pathologic examination under routine conditions. On the other hand, in many ruminant cases, only CNS tissues were available, as they were investigated as part of bovine spongiform encephalopathy statutory disease surveillance, which targets brain tissues. In that situation, other tissues as well as body fluids are no longer accessible at the time of diagnosis. A systematic sampling and testing plan of animals with neurological disease would significantly advance our knowledge of disease pathogenesis.

One of the main obstacles in astrovirus research is the absence of functional cell culture systems. To date, among members of the genus *Mamastrovirus*, only classical human astroviruses (*MAstV 1*) [79], individual bovine astroviruses [80,81], feline astroviruses [82], porcine astroviruses [83] and the HuAstV VA1 strain [70,84] have been propagated in cell culture successfully. In addition, reverse genetic systems, which are a prerequisite for functional studies on the molecular biology and host interactions of these viruses, have been established only for HuAstV VA1/HMO-C and classical human astroviruses [70,85], and none is available for animal NT-AstVs. 

The cases of PoAstV-3 and MiAstV-associated encephalitis underpin the importance of an epidemiological assessment of the disease situation. These disease outbreaks exemplify that NT-AstV infection can reach endemic—potentially epidemic—proportions with consequences for animal health and livestock economy. Data also suggest that astroviruses can cross the species barrier and are capable of genetic recombination, rapid evolution and adaption to different host species [52,86,87,88]. Together with the fact that astroviruses are found almost worldwide and can be detected in a broad spectrum of vertebrates [14], their zoonotic significance cannot be ignored. In this regard, astroviruses have remained neglected over the decades, and the possibility that astroviruses could cross the species barrier from animals to humans, or vice versa, should be the driving force for future research into astrovirus-associated diseases.
